# Photocatalytic Degradation of Ceftriaxone Using TiO_2_ Coupled with ZnO Micronized by Supercritical Antisolvent Route

**DOI:** 10.3390/nano13243130

**Published:** 2023-12-13

**Authors:** Antonietta Mancuso, Stefania Mottola, Olga Sacco, Vincenzo Vaiano, Iolanda De Marco

**Affiliations:** 1 Department of Industrial Engineering, University of Salerno, Via Giovanni Paolo II 132, 84084 Fisciano, Italy; anmancuso@unisa.it (A.M.); smottola@unisa.it (S.M.); idemarco@unisa.it (I.D.M.); 2 Department of Chemistry and Biology “A. Zambelli”, University of Salerno, Via Giovanni Paolo II 132, 84084 Fisciano, Italy; osacco@unisa.it

**Keywords:** supercritical antisolvent, composite photocatalyst, ZnO/PC50, degradation, photocatalysis, ceftriaxone

## Abstract

Heterogeneous photocatalysis is a promising technique for removing pollutants from water. In this work, supercritical antisolvent (SAS)-micronized ZnO (ZnO_SAS_) is coupled with commercial anatase TiO_2_ (PC50) to study the photocatalytic degradation of ceftriaxone under UV and visible light. Diffuse ultraviolet–visible reflectance (UV−vis DRS) measurement revealed that the presence of ZnO leads to a slight absorption in the visible region. Wide-angle X-ray diffraction (WAXD) analysis showed the presence of both ZnO wurtzite and TiO_2_ anatase crystalline phases in the composite. Photocatalytic tests proved that the activity of the ZnO_SAS_/PC50 composite is higher than that of commercial ZnO, SAS-micronized ZnO, and PC50, allowing complete ceftriaxone degradation under UV light after only 2 min of irradiation time. In contrast, about 90% of ceftriaxone degradation is achieved after 180 min of visible-light irradiation. The photocatalytic results for an experiment carried out in the presence of probe scavenger molecules for reactive oxygen species show that hydroxyl radicals and positive holes are both reactive species involved in the ceftriaxone photocatalytic degradation mechanism. Finally, reuse cycles of the ZnOsas/PC50 composite are performed, demonstrating the stability and recyclability of the photocatalyst.

## 1. Introduction

Population growth and the development of industries favored by technological and scientific progress have led to an increase in water pollution. In particular, the growing contamination of wastewater by organic and inorganic pollutants refractory to conventional treatment methods requires the development of innovative technologies capable of degrading them. Advanced oxidation processes (AOPs) are efficient methods for achieving the degradation of numerous organic substances. AOPs are based on generating highly reactive species, mainly hydroxyl radicals (·OH), which can oxidize pollutants into non-harmful substances. Among AOPs, heterogeneous photocatalysis using semiconductor oxides has proven to be a very effective solution for removing pollutants [1,2]. Both zinc oxide (ZnO) and titanium dioxide (TiO_2_) are n-type semiconductors widely used as photocatalysts for the removal of several water pollutants [3,4,5,6,7]. However, the practical applications of these two semiconductors are limited by their large band gap (3.37 eV for ZnO [8] and 3.2 eV for TiO_2_ [9]), which makes them able to absorb only UV light, and by the rapid recombination of photogenerated holes and electrons [10,11]. Hence, these semiconductors cannot use the potential of solar photocatalysis. Therefore, several techniques have been employed to allow the absorption of photons with lower energy. These techniques include surface modification [12,13,14], band gap modification by doping with metals and non-metals [15,16,17,18], and semiconductor coupling [19,20]. In general, to suppress the recombination phenomena of photoproduced electron–hole pairs and extend the absorption range of semiconductors into the visible-light region [21], a promising and interesting strategy is the manufacture of composites obtained by coupling two semiconductors [20,22,23,24]. From this perspective, the study and engineering of composites is one of the approaches most widely adopted by researchers to obtain an improvement in the photocatalytic activity [19,25,26,27,28]. For instance, Marcì et al. formulated ZnO/TiO_2_ heterojunctions that showed an enhancement in photocatalytic activity for the phenol, 2-chlorophenol, and pentachlorophenol degradation compared to bare TiO_2_ and ZnO [19]. Mousa et al. synthesized TiO_2_/ZnO heterojunctions for dye photodegradation [21]. They proved that the coupling of ZnO and TiO_2_ led to an increase in the lifetime of the photogenerated charges, allowing more efficient separation of electron–hole pairs due to the electron transfer from the conduction band of ZnO to the conduction band of TiO_2_ with the simultaneous transfer of positive holes from the valence band of TiO_2_ to the valence band of ZnO [21]. In recent years, ZnO-based photocatalysts in the form of nanoparticles were prepared by the supercritical antisolvent (SAS) precipitation route [29,30,31,32]. They showed better photocatalytic performances than those prepared by drying–precipitation and sol–gel methods [32]. Indeed, using Eu-doped ZnO prepared by SAS precipitation, it was possible to remove Eriochrome Black-T dye under UV and visible irradiation. The SAS-prepared sample showed better photocatalytic efficiency in terms of both discoloration and mineralization than the drying–precipitation sample. In particular, the SAS-prepared sample reached almost total dye removal after 240 min of UV light irradiation, whereas for the sample prepared via drying–precipitation, the total organic carbon (TOC) reduction was equal to 80% [32].

Considering these promising results, in this preliminary experimental work, ZnO/TiO_2_ composites were synthesized using commercial ZnO, ZnO micronized through the supercritical antisolvent technique, and commercial TiO_2_ to enhance the photocatalytic performance of bare ZnO and TiO_2_.

The photocatalytic activity of the composites was evaluated using ceftriaxone as a model pollutant in the presence of UV light and visible-light irradiation.

## 2. Materials and Methods

### 2.1. Materials

Zinc acetate dihydrate (C_4_H_6_O_4_Zn·2H_2_O, purity ≥ 99%) and commercial ZnO (named ZnO_comm_) were provided by Carlo Erba (St. Louis, MO, USA), and commercial TiO_2_ (PC50) was produced by Cristal Global Millennium Inorganic Chemical. Dimethylsulfoxide (DMSO, purity 99.8%) was supplied by Carlo Erba. CO_2_ (purity 99%) was provided by Morlando Group s.r.l. (Naples, Italy). Ceftriaxone sodium (C_18_H_16_N_8_Na_2_O_7_S_3_ 3.5H_2_O) was purchased from Merk Generics. Isopropyl alcohol (CH_3_CH(OH)CH_3_) was provided by Merk Life Science S.r.l. (Milan, Italy). Distilled water was used as a solvent to prepare ceftriaxone solutions.

### 2.2. Preparation of ZnO from ZnAc Micronized through Supercritical Antisolvent (SAS) Technique

The micronization of zinc acetate dihydrate (ZnAc) powders was carried out using the SAS process [32]. This process exploits the peculiar characteristics of carbon dioxide in the supercritical state: diffusivities comparable to those of gases and densities of an order of magnitude similar to those of liquids [29].

The SAS plant comprises a cylindrical vessel with an internal volume of 500 cm^3^, serving as the core of the process. To attain the required pressure, two high-pressure pumps were employed to introduce CO_2_ (antisolvent) and a liquid solution (DMSO + solute) into the chamber. CO_2_ was pre-cooled in a refrigerated bath before being introduced into the chamber, while the liquid solution was injected into the precipitation chamber through a stainless-steel nozzle. A Proportional Integral Derivative (PID) controller, coupled with heating bands, was used to maintain the vessel’s operating temperature. Pressure regulation was achieved via a micrometric valve, and pressure measurement was facilitated by a test gauge manometer. At the bottom of the precipitation chamber, a porous filter with 0.1 μm diameter pores allowed the passage of the CO_2_-solvent mixture, enabling the collection of the precipitated powders. The flow rate of CO_2_ was measured using a rotameter. At the beginning of each SAS experiment, CO_2_ was pumped into the precipitation chamber until the desired pressure and temperature were achieved.

**Figure 1:**
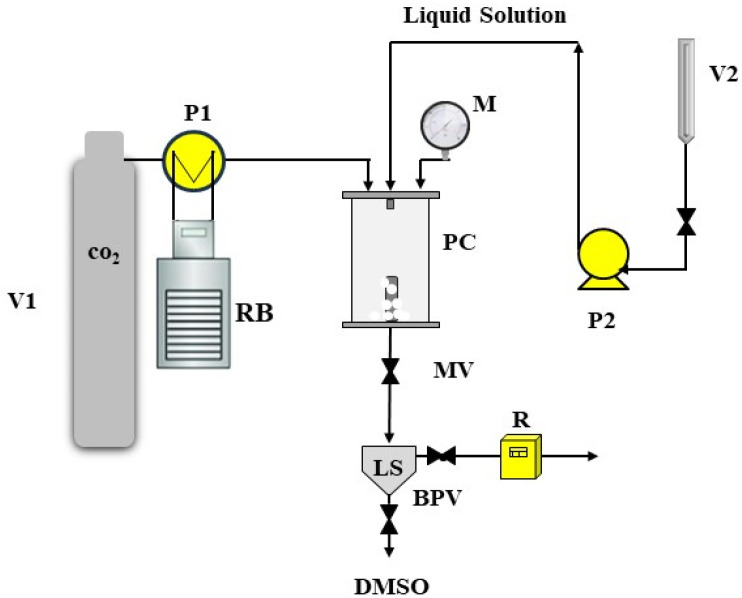
SAS plant sketch. V1: carbon dioxide tank; V2: liquid solution burette; RB: refrigeration bath; P1: carbon dioxide pump; P2: liquid pump; PC: precipitation chamber; M: manometer; MV: micrometric valve; LS: liquid separator; BPV: back-pressure valve; R: rotameter; DMSO: outcoming solvent.

Once the quasi-steady-state composition of solvent and antisolvent was established, the liquid solution was introduced through the nozzle to initiate solute micronization. After the solution injection, CO_2_ continued to flow for a calculated duration to ensure the complete removal of solvent residues. Subsequently, the CO_2_ pump was turned off, and the vessel was depressurized gradually to atmospheric pressure. In our experiments, the liquid flow rate was set at 1 mL min^−1^, and the CO_2_ flow rate was 30 g min^−1^ for ZnAc. A schematic representation of SAS plant is shown in Figure 1.

Micronization conditions, including a pressure equal to 150 bar, temperature equal to 40 °C, and concentration of 15 mg mL^−1^ for the micronization of ZnAc, were chosen following a previous work [29]. Before each experiment, the solution was prepared by dissolving the solute in 100 mL of DMSO, which served as the solvent, to achieve the desired concentration and maximum sample yield. After each SAS experiment, the powder was collected in the precipitator, and DMSO was extracted using supercritical carbon dioxide (scCO_2_). The obtained powder was annealed at 500 °C for 2 h in air with a heating rate equal to 2 °C min^−1^ to obtain zinc oxide nanoparticles (named ZnOSAS) [29].

### 2.3. Preparation of ZnO/PC50 Composites

The preparation of the semiconductor–semiconductor composites was performed through mechanical mixing involving the use of isopropanol in aqueous medium [33]. In detail, 0.176 g of ZnO (ZnO_comm_ or ZnO_SAS_) and 1 g of commercial TiO_2_ (PC50) powder were added to an aqueous solution (100 mL) of isopropanol (1 M). The suspension was subsequently maintained at 80 °C under continuous stirring to allow solvent removal and obtain ZnO_comm_/PC50 and ZnO_SAS_/PC50 composites. The content of ZnO in both composite samples was 15 wt%, which was optimized in a previous paper [33].

The samples are named as follows:

- PC50;
- ZnO_comm_;
- ZnO_SAS_;
- ZnO_comm_/PC50;
- ZnO_SAS_/PC50.

### 2.4. Characterization Methods

A field emission scanning electron microscope (FESEM, mod. LEO 1525, Carl Zeiss SMT AG, Oberkochen, Germany) was employed to detect the morphology of the samples; the powders collected in the chamber for each test were distributed on a carbon tab (Agar Scientific, Stansted, UK) and enclosed with gold–palladium (layer thickness 250 Å) using a sputter coater (mod. 108 A, Agar scientific, Monterotondo, Italy). The diffuse ultraviolet–visible reflectance (UV-Vis DRS) spectra of the samples, recorded with an RSA-PE-20 reflectance spectroscopy accessory (Labsphere Inc., North Sutton, NH, USA), were obtained using a Perkin Elmer Lambda 35 spectrophotometer (Waltham, MA, USA). The band gap values were calculated through the corresponding Kubelka–Munk function (F(R_∞_)) and by plotting [F(R_∞_)∙hν]^2^ against hυ (eV). The Brunauer, Emmett, and Teller (BET) surface area of the samples was measured by dynamic N_2_ adsorption measurement at −196 °C using a Costech Sorptometer 1042 (Costech International S.p.A., Milan, Italy); all the samples before the measurement were pretreated at 150 °C for 30 min in He flow. Wide-angle X-ray diffraction (WAXD) patterns were obtained with an automatic Bruker D8 Advance diffractometer (VANTEC-1 detector, Milan, Italy) using nickel-filtered Cu-Kα radiation. Laser Raman spectra were achieved at room temperature with a Dispersive MicroRaman (Invia, Renishaw) equipped with a 514 nm laser in the range of 100–2000 cm^−1^ Raman shift.

### 2.5. Photocatalytic Activity Tests

The photocatalytic activity tests were carried out under UV light and visible light. The tests were performed using 0.02125 g of PC50 (dosage = 0.425 g L^−1^) and 0.00375 g of ZnO_comm_ and ZnO_SAS_ (dosage = 0.075 g L^−1^) corresponding to the amounts of PC50 and ZnO present in composites and 0.025 g of ZnO_comm_/PC50 and ZnO_SAS_/PC50 (dosage = 0.5 g L^−1^). The photocatalysts were dispersed in 50 mL of ceftriaxone aqueous solution with an initial concentration of 5 mgL^−1^. The entire duration of the UV-light-driven tests was 10 min: 60 min in the dark phase and 10 min under UV light, whereas the visible-light-driven tests had an overall duration of 240 min: 60 min in the dark phase and 180 min under visible light. The suspension was introduced into a cylindrical Pyrex reactor (ID = 3 cm and V_TOT_ = 200 mL, Microglass Heim Srl, Naples, Italy). UV-LEDs (nominal power 10W, provided by Daylight White Light, Shenzen, China) emitting at 365 nm and visible LED (nominal power 10W, provided by Daylight White Light, China strips) strips with emissions in the 400–800 nm wavelength range were placed around the reactor to irradiate the suspensions. The photoreactor was placed on a magnetic stirrer, and the air was bubbled inside the suspension to prevent the sedimentation of the photocatalyst particles. Liquid samples were taken during the test time using a syringe with a volume of 3 mL, which were then filtered and placed in 2 mL vials.

High-Performance Liquid Chromatography (HPLC) (Agilent Technologies 1200 infinity series, Santa Clara, CA, USA) was employed to measure the ceftriaxone concentration. A C18 as a stationary phase column operating on isocratic elution using a mobile phase constituted by a mixture of 80:20, KH_2_PO_4_ buffer, and methanol, was used [29].

The removal efficiency of ceftriaxone was determined with the following formula:

(1) $$\eta =\left(1-\frac{C}{C_{0}}\right)•100$$

where C is the ceftriaxone concentration at the generic irradiation time, and $C_{0}$ is the initial ceftriaxone concentration.

## 3. Results and Discussion

### 3.1. Chemical–Physical Characterization of Photocatalysts

#### 3.1.1. FESEM Characterization

**Figure 2:**
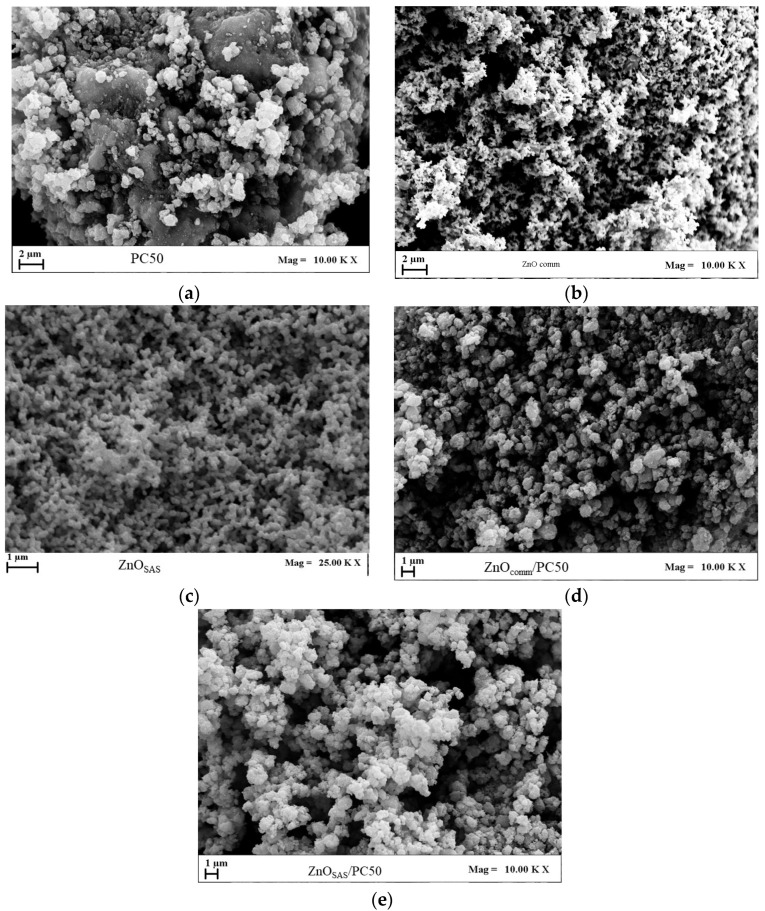
FESEM images of all the commercial samples produced with the SAS technique and composite samples: (**a**) commercial PC50, (**b**) commercial ZnO, (**c**) ZnO produced with the SAS technique, (**d**) composite sample produced using commercial ZnO, and (**e**) composite sample produced using ZnO with the SAS technique.

The images in Figure 2 show the morphologies of different samples: the commercial samples produced with the SAS technique and composite samples. Figure 2a,b show the SEM micrography of commercial samples; it is evident that these samples had crystalline and irregular shapes. Observing Figure 2c, it is clear that using the SAS micronization allowed for obtaining amorphous nanoparticles of ZnO with a mean diameter of 0.08 µm, as reported in Table 1. The obtained composites (Figure 2d,e) exhibited a predominantly cohesive structure. This cohesion is likely attributed to the sample preparation method, which includes mixing in the presence of isopropanol and a drying stage at room temperature, potentially resulting in particle aggregation.

The data on morphology, mean diameter (md), and standard deviation (sd) are reported in Table 1.

#### 3.1.2. UV-vis DRS Results

**Figure 3:**
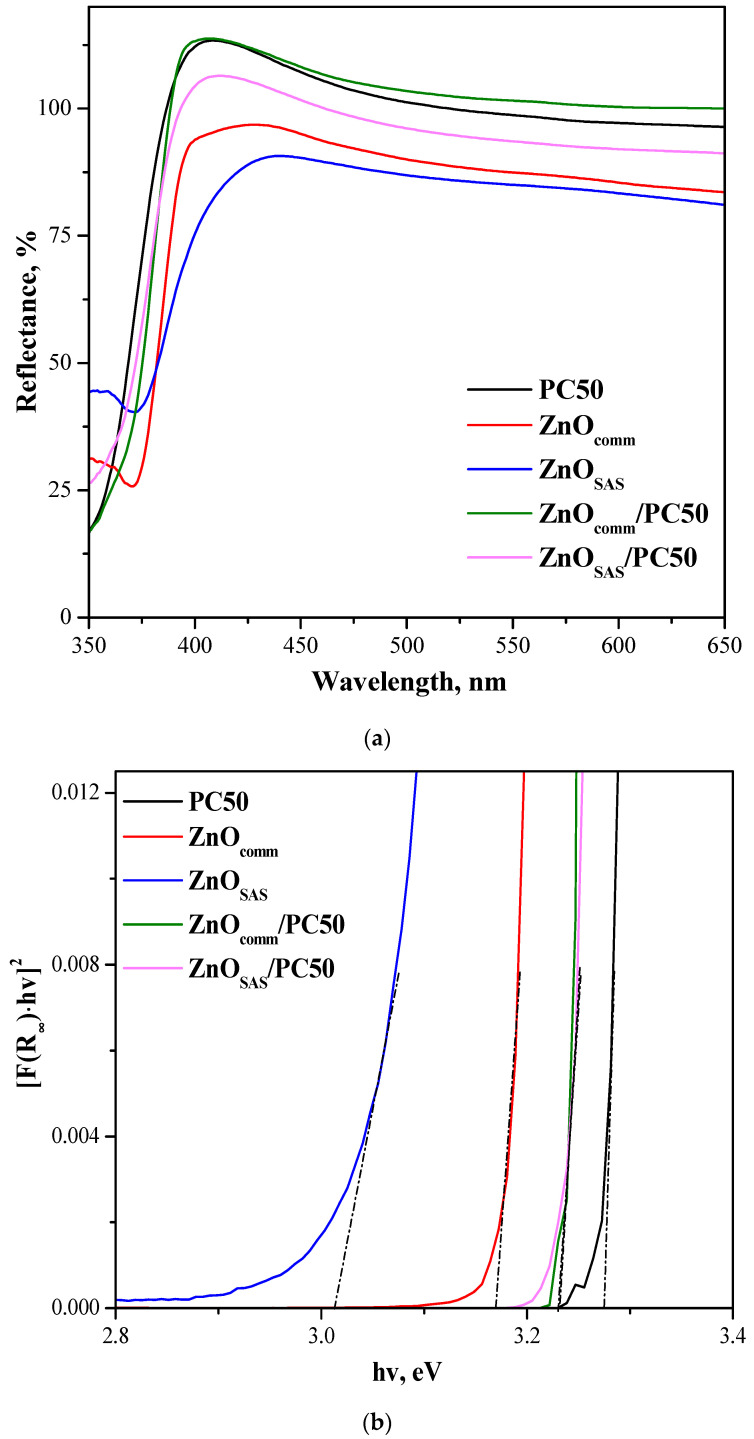
(**a**) Reflectance spectra of PC50, ZnO_comm_, ZnO_SAS_, ZnO_comm_/PC50, and ZnO_SAS_/PC50 samples; (**b**) plot of [F(R_∞_) hv]^2^ versus the photon energy of PC50, ZnO_comm_, ZnO_SAS_, ZnO_comm_/PC50, and ZnO_SAS_/PC50 samples. The intersection of the dashed lines with the x-axis gives the value of E_g_.

The UV-vis DRS spectra in the wavelength range of 350–650 nm for all the photocatalysts are shown in Figure 3a.

PC50, ZnO_SAS_, and ZnO_comm_ showed strong absorption in the UV region (λ < 400 nm), but, as expected, no absorption was found in the visible region. The same result was observed for ZnO_comm_/PC50 and ZnO_SAS_/PC50. In addition, the absorption band of ZnO_comm_ and ZnO_SAS_ located at about 360 nm disappeared for both composites because of the shielding effect of TiO_2_ in the UV-light region [34]. Band gap values (E_g_) were calculated using the Kubelka–Munk function (Figure 3b), and the obtained values are reported in Table 1. The E_g_ was 3.27 and 3.17 eV for PC50 and ZnO_comm_, while the lowest value of E_g_ (3.03 eV) was measured for ZnO_SAS_, revealing a slight red shift of absorption onset in the visible region (at about 410 nm). However, a slight increase in E_g_ was observed for both composites concerning the E_g_ value of ZnO_SAS_. In particular, the same E_g_ value of about 3.22 eV was obtained for ZnO_comm_/PC50 and ZnO_SAS_/PC50 systems.

#### 3.1.3. WAXD Results

**Figure 4:**
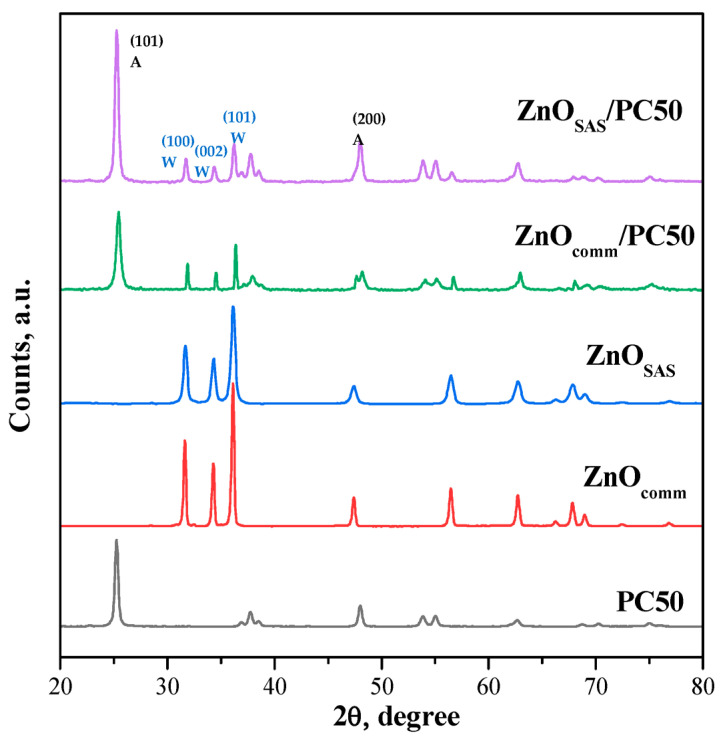
WAXD patterns of PC50, ZnO_comm_, ZnO_SAS_, ZnO_comm_/PC50, and ZnO_SAS_/PC50 samples; W = wurtzite; A = anatase.

The wide X-ray diffraction (WAXD) patterns of all photocatalysts are shown in Figure 4. The PC50 sample showed the main diffraction signals typical of titania in the anatase phase with two main reflexes at 2θ = 25.3° and 48°, which are referred to as the diffraction crystalline faces (101) and (200), respectively [35,36]. The diffraction patterns of ZnO_comm_ and ZnO_SAS_ displayed the characteristic reflections of the wurtzite phase with the main peaks located at 2θ = 31.9°, 34.5°, and 36.3°, being ascribed to the (100), (002), and (101) faces, respectively [36]. The WAXD spectra of the ZnO_comm_/PC50 and ZnO_SAS_/PC50 composites confirmed the presence of both the wurtzite and anatase phases of ZnO and PC50, respectively. Moreover, the crystallite sizes (D) of the wurtzite and anatase phases identified in both the composites (Table 1) were very similar to those of ZnO_comm_, ZnO_SAS_, and PC50, proving that, in agreement with the literature, the coupling of the two semiconductors realized through the isopropanol method did not modify the crystalline phases of bare ZnO_comm_, ZnO_SAS_, and PC50 [33].

#### 3.1.4. Specific Surface Area (SSA) Measurement

The SSA values estimated using the BET method are reported in Table 1. PC50 showed a high value of 44 m^2^g^−1^ according to the literature [33], whereas the SSA data of ZnO_comm_ and ZnO_SAS_ are lower than those observed for PC50, being equal to 6 and 20 m^2^g^−1^, respectively. The lower value of SSA for ZnO_comm_ is in agreement with the values reported in the literature [37,38]. The SSA value of composites was very similar to the SSA value of PC50, probably due to the high amount of PC50 in the photocatalytic composites. Also, the SSA data of ZnO/PC50 composites agree with the values reported in the literature [33,39]. In particular, ZnO_comm_/PC50 and ZnO_SAS_/PC50 samples evidenced a similar SSA value of approximately 40 m^2^g^−1^, which is higher than that obtained for bare ZnO_comm_ and ZnO_SAS_.

#### 3.1.5. Raman Spectroscopy Results

**Figure 5:**
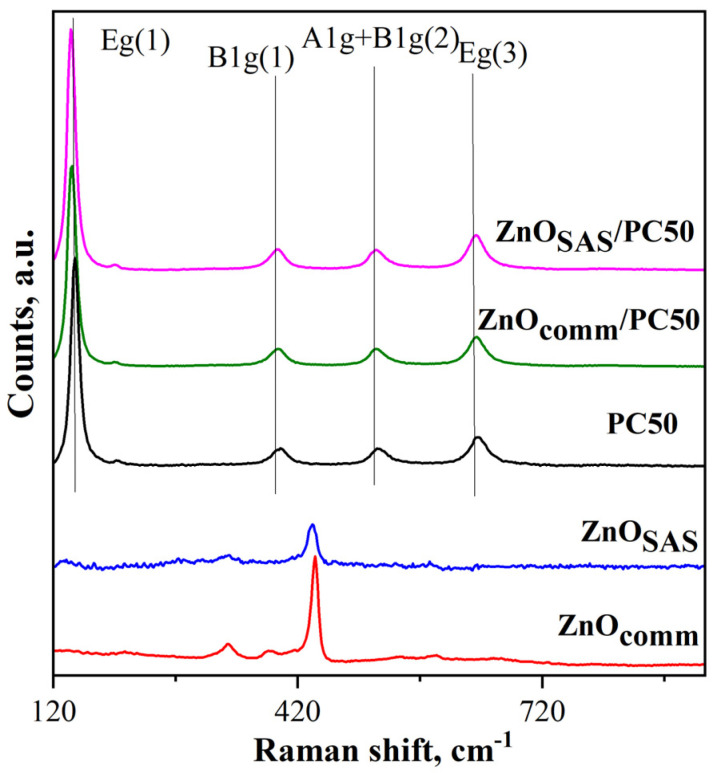
Raman spectra of PC50, ZnO_comm_, ZnO_SAS_, ZnO_comm_/PC50, and ZnO_SAS_/PC50 photocatalysts.

Raman analysis was carried out at room temperature, and the photocatalysts’ spectra in the range 100–900 cm^−1^ are displayed in Figure 5.

The Raman bands for PC50 were observed at 144, 192, 393, 513, and 636 cm^−1^, corresponding to the Eg(1), Eg(2), B1g(1), A1g + B1g(2), and Eg(3) of anatase modes, respectively [35]. The Raman signals observed for ZnO_comm_ and ZnO_SAS_ were ascribed to the active modes of wurtzite ZnO crystal [40]. In particular, the E2 (high) Raman mode observed at about 437 cm^−1^ and at 332 cm^−1^, ascribed to E_2_(H)–E_2_(L) (multi-phonon), was identified [41,42]. In agreement with the literature [34], only Raman bands of anatase TiO_2_ were found in ZnO_comm_/PC50 and ZnO_SAS_/PC50. Still, the signals of ZnO in the composites were not detected due to the higher percentage of TiO_2_ in ZnO_comm_/PC50 and ZnO_SAS_/PC50 photocatalysts. However, a blue shift of the Eg(1) Raman active mode of PC50 from 144 to about 142 cm^−1^ was observed for the two composites. This blue shift could be due to the presence of oxygen vacancies in the ZnO_comm_/PC50 and ZnO_SAS_/PC50 photocatalysts [43,44].

### 3.2. Photocatalytic Activity Results

**Figure 6:**
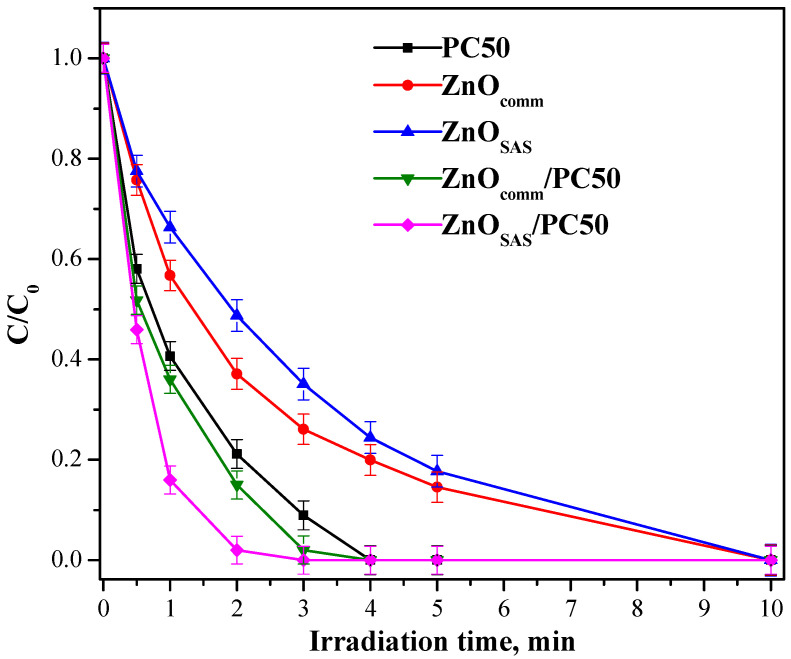
Photocatalytic tests of ceftriaxone using PC50, ZnO_comm_, ZnO_SAS_, ZnO_comm_/PC50, and ZnO_SAS_/PC50 photocatalysts in the presence of UV light (wavelength equal to 365 nm).

Figure 6 shows the degradation of ceftriaxone under UV light (wavelength equal to 365 nm) using PC50, ZnO_comm_, ZnO_SAS_ s, ZnO_comm_/PC50, and ZnO_SAS_/PC50.

**Figure 7:**
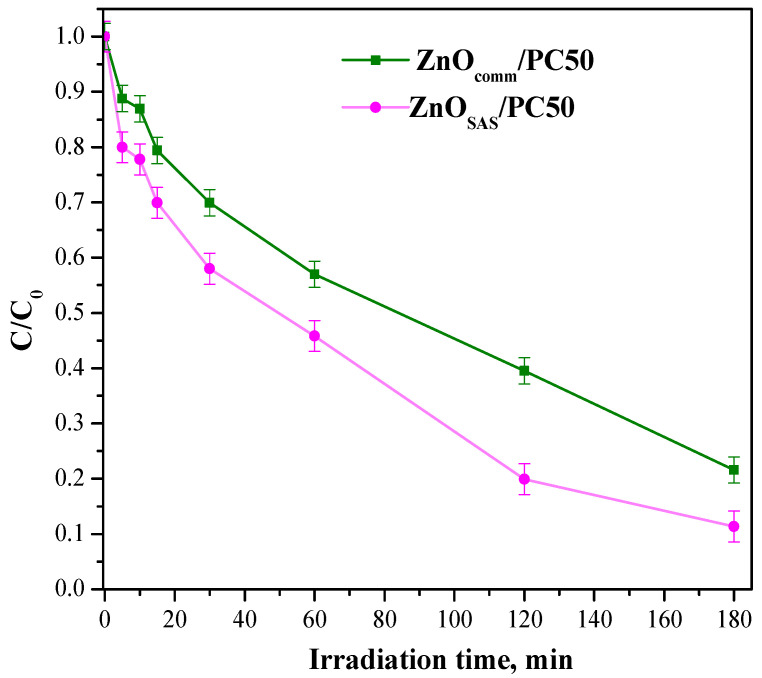
Photocatalytic tests of ceftriaxone using ZnO_comm_/PC50 and ZnO_SAS_/PC50 composites in the presence of visible-light irradiation (wavelength in the range 400–800 nm).

The bare ZnO_comm_ and ZnO_SAS_ samples completely degraded ceftriaxone after 10 min of UV light, while PC50 showed a degradation efficiency higher than both ZnO_comm_ and ZnO_SAS_. Indeed, in the presence of PC50, complete ceftriaxone degradation was achieved after 4 min of irradiation time. In the presence of both composites, an increased ceftriaxone degradation rate was observed, probably because the charge–carriers recombination phenomenon was inhibited [21,36]. Also, in the case of other pollutants, such as ciprofloxacin, the photocatalysis with modified TiO_2_ nanocrystals gave good results [45]. In this paper, the ZnO_SAS_/PC50 composite allowed the complete elimination of ceftriaxone after only 2 min of irradiation. The formulated ZnO_comm_/PC50 and ZnO_SAS_/PC50 composites were also tested in ceftriaxone degradation under visible irradiation (wavelength in the range 400–800 nm) (Figure 7).

Under visible-light irradiation, the ZnO_SAS_/PC50 composite exhibited a higher degradation rate in the overall reaction time concerning ZnO_comm_/PC50. In particular, photocatalytic degradation efficiency was about 90% in the presence of ZnO_SAS_/PC50 after 180 min of visible light, which can probably be attributed to the presence of the ZnO_SAS_ photocatalyst that can absorb visible light to a certain extent (see Figure 3).

The ceftriaxone degradation follows the pseudo-first-order kinetics [29,46,47] defined by the following equation:

(2) $$-ln\frac{C}{C_{0}}=k•t$$

The kinetics constant value was the straight-line slope derived by plotting $-ln\frac{C}{C_{0}}\;$ vs. the irradiation time.

Table 2 reports the degradation constant values under UV and visible-light irradiation.

ZnO_SAS_/PC50 exhibited the highest degradation rate in the presence of UV light with a kinetic constant equal to 2.00 min^−1^, proving the improved activity related to the coupling of two semiconductors. Moreover, the ZnO_SAS_/PC50 composite showed a higher degradation rate (~0.0131 min^−1^) than that observed with ZnO_comm_/PC50 (~0.0085 min^−1^), even under visible-light irradiation.

#### 3.2.1. Role of Reactive Oxygen Species in Ceftriaxone Photocatalytic Degradation in the Presence of the ZnO_SAS_/PC50 Composite

The influence of reactive oxygen species (ROS) in ceftriaxone photocatalytic degradation was studied using the ZnO_SAS_/PC50 composite. Ethylenediaminetetraacetic acid (EDTA, 10 mM), isopropanol (IPA, 10 mM), and benzoquinone (BQ, 1 μM) were added as scavenger probe molecules to quench positive holes [15], hydroxyl radicals [48], and superoxide [49].

**Figure 8:**
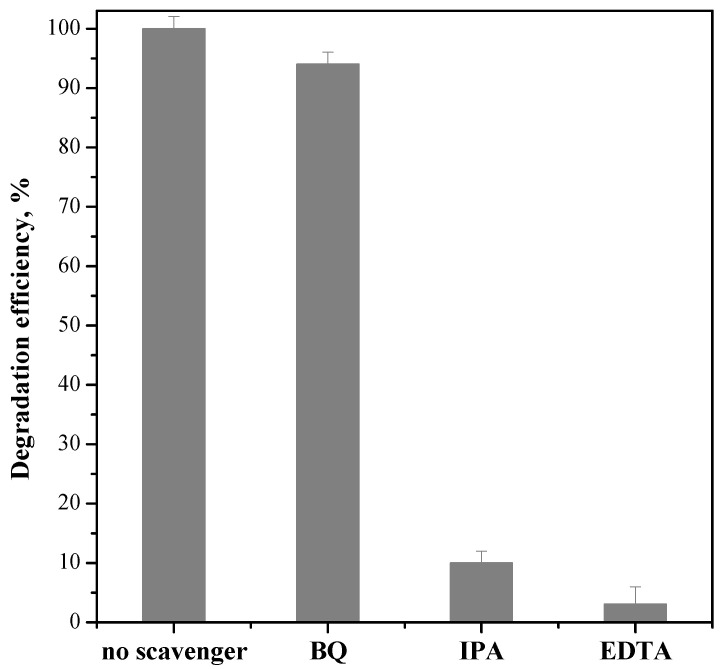
Effect of ethylenediaminetetraacetic acid (EDTA), isopropanol (IPA), and benzoquinone (BQ) on ceftriaxon degradation using ZnO_SAS_/PC50 composite under UV light.

Figure 8 shows that adding EDTA, IPA, and BQ led to variation in the degradation efficiency of ceftriaxone under UV light irradiation. In particular, the presence of EDTA and IPA significantly reduced the ceftriaxone photodegradation performances, underlining that the positive holes and hydroxyls are the main ROS involved in the degradation mechanism of ceftriaxone driven by UV irradiation.

#### 3.2.2. Possible Mechanism of the Photocatalytic Activity of the ZnO_SAS_/PC50 Composite

A possible mechanism of the activation of the ZnO_SAS_/PC50 composite under UV light was proposed based on the results of the photocatalytic degradation of ceftriaxone under UV irradiation carried out in the presence of scavengers (Figure 8), which demonstrate that hydroxyl radicals and positive holes are the main ROS involved in ceftriaxone degradation. Considering this, the Mulliken relationship was used for calculating the edge position of the conduction band (E_CB_) and valence band (E_VB_) for ZnO_SAS_ and PC50 [50]:

(3) $$E_{CB}=X-E_{e}-0.5{\;*\;E}_{bg}$$

(4) $$E_{VB}={E_{CB}+E}_{bg}$$

where X is the absolute electronegativity of ZnO (5.94 eV) and TiO_2_ (5.81 e V) [51,52], E_e_ is the energy of free electrons on the hydrogen scale (ca. 4.5 eV), and E_bg_ is the band gap energy of the ZnO_SAS_ (3.03 eV) and PC50 (3.27 eV) obtained from UV-vis DRS measurement (Table 1).

**Figure 9:**
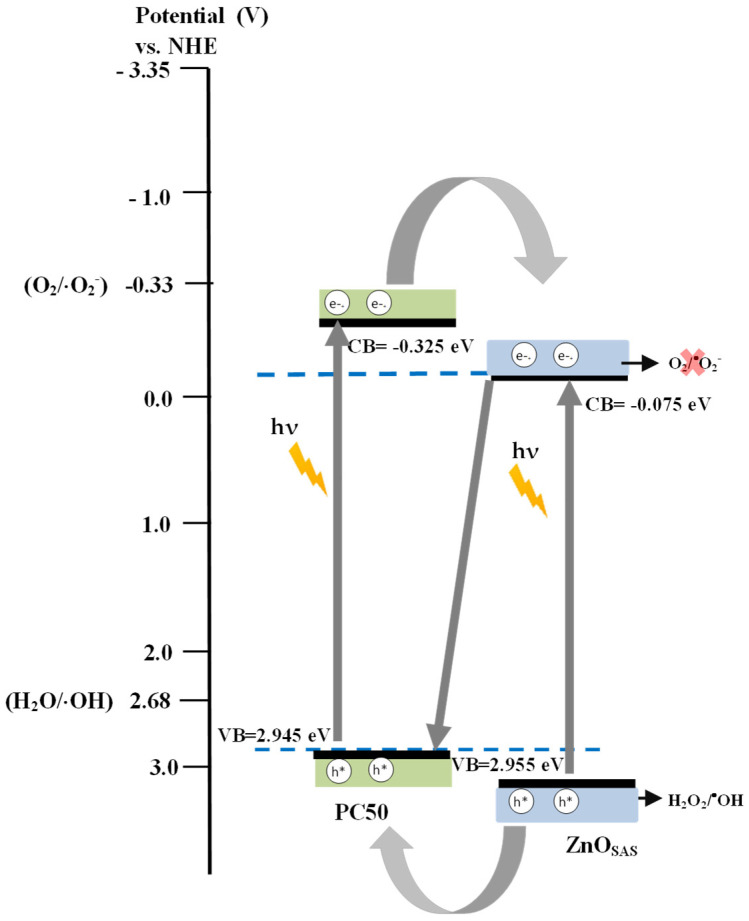
Proposed mechanism for electron and hole transfer in ceftriaxone degradation under UV light irradiation.

Based on the literature [33,53,54] and considering the results obtained, a Z-scheme composite was proposed (Figure 9).

In the presence of UV light, the photogenerated holes migrated from the VB of ZnO_SAS_ to the VB of PC50, whereas the electrons promoted in the CB of PC50 transferred into the CB of ZnO_SAS_. However, since E_CB_ of ZnO_SAS_ (−0.075 eV) was less negative than the standard reduction potential of O_2_/·O_2_^−^ (−0.33 eV vs. NHE), the electrons in CB of ZnO_SAS_ were not able to reduce oxygen into reactive ·O_2_^−^. Therefore, at the interface between the two semiconductors, the electrons CB of ZnO_SAS_ were free to migrate into the VB of PC50 [55]. This transfer mechanism maintains the photogenerated charge carriers separated on opposite side of the composite, reducing the charge carriers’ recombination rate [33]. On the other hand, the E_VB_ of PC50 (+2.945 eV vs. NHE) was more positive than that of ·OH/H_2_O (+2.68 eV vs. NHE), and therefore the positive holes could react with H_2_O to form ·OH. When the positive holes were quenched by adding the EDTA scavenger (Figure 8), they were not available to generate ·OH. Therefore, this suggested activation mechanism agrees with the experimental results on the role of ROS reported in Figure 8, which shows that OH and h^+^ acted in ceftriaxone photocatalytic degradation under UV irradiation. Finally, the photocatalytic degradation activity of ZnO_SAS_/PC50 in the presence of visible light (Figure 7) could be explained by the interface gap between the CB of PC50 (+2.945 eV vs. NHE) and VB of ZnO_SAS_ (−0.075 eV vs. NHE), which allowed the activation of the photocatalyst under visible light [33].

#### 3.2.3. Stability Tests on ZnO_SAS_/PC50 Composite

**Figure 10:**
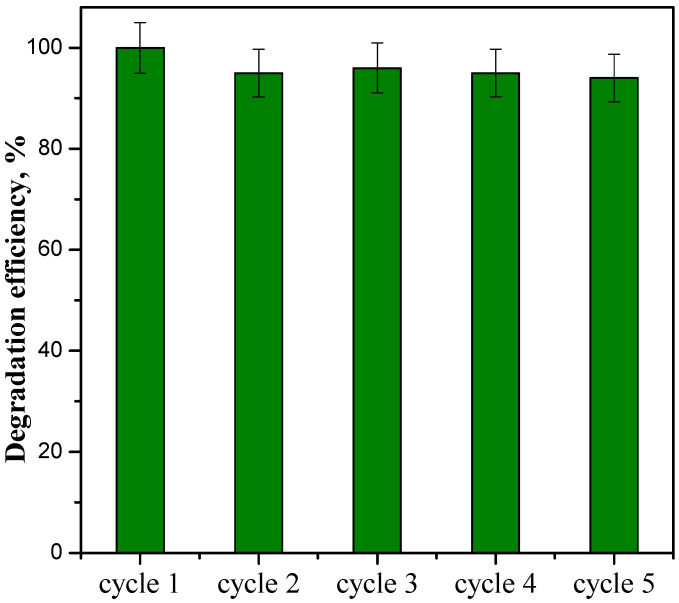
Reuse cycles of the ZnO_SAS_/PC50 composite.

Different reuse cycles of ceftriaxone degradation under UV light were performed to investigate the stability of the ZnO_SAS_/PC50 composite, which showed the best photocatalytic performance. In detail, 0.5 g L^−1^ of catalyst dosage, an initial ceftriaxone concentration of 5 mg L^−1^, and a volume of the solution equal to 50 mL were employed. The photocatalyst was recovered after each test, subsequently centrifuged, washed with distilled water, and dried at room temperature for 24 h. Figure 10 shows the ceftriaxone degradation efficiency after 10 min of UV light in five recycling tests.

From the first to the fifth cycle, no substantial reduction in the degradation efficiency of ceftriaxone was found in the presence of UV light, demonstrating that ZnO_SAS_/PC50 is a very stable catalyst and can be reused several times without losing its original catalytic activity.

## 4. Conclusions

A SAS-micronized ZnO photocatalyst (ZnO_SAS_) and commercial ZnO (ZnO_comm_) were coupled with commercial TiO_2_ (PC50) by mechanical mixing in an aqueous phase with the addition of isopropanol. FESEM images showed the regular shape of the SAS-obtained sample. WAXD patterns evidenced the presence of wurtzite and anatase crystalline phases by confirming the incorporation of the two semiconductor materials in the ZnOcomm/PC50 and ZnOSAS/PC50 samples, resulting in the creation of a composite. The activity results under UV light demonstrated that coupling the two semiconductors led to higher photocatalytic activity than just using the bare samples. In particular, the ZnO_SAS_/PC50 composite achieved a complete removal of ceftriaxone after just 2 min of irradiation time. Photocatalytic experiments in the presence of UV light performed with the addition of the probe scavenger molecules showed that the main reactive oxygen species responsible for the ceftriaxone degradation mechanism were the positive holes and hydroxyl radicals. Based on such results and considering the UV-Vis DRS findings, the Mulliken equation was used to propose a possible scheme of the band alignment together with the photogenerated charge–transfer mechanism for the ZnO_SAS_/PC50 composite under UV light. The results evidence the possible formation of a Z-scheme heterojunction that facilitates the transport of photogenerated electrons at the interface between the two semiconductors, reducing the e–/h+ recombination rate and improving the photocatalytic degradation performances. Moreover, ZnO_SAS_/PC50 also exhibited an appreciable degradation activity under visible light with a degradation efficiency of about 90% after 180 min of visible light, probably due to the generation of an interface gap between the conduction band of PC50 and the valence band of ZnO. Finally, the stability and reusability of the ZnO_SAS_/PC50 composite were verified after five recycling cycles.

## Figures and Tables

**Table 1:** Morphology, mean diameter (md), standard deviation (sd), crystallite size, specific surface area (SSA), and band gap (E_g_) values of all synthesized samples. (NP = nanoparticle; cMP = coalescent microparticle; C = crystal).

Sample	Morphology	md ± sd [μm]	D [nm] Anatase Wurtzite	SSA [m^2^g^−1^]	E_g_ [eV]
PC50	C	-	26	44	3.27
ZnO_comm_	C	-	24	6	3.17
ZnO_SAS_	NP	0.08 ± 0.007	22	20	3.03
ZnO_comm_/PC50	cMP	-	25 25	40	3.22
ZnO_SAS_/PC50	cMP	-	27 23	39	3.22

**Table 2:** Apparent degradation kinetic constant (k) using PC50, ZnO_comm_, ZnO_SAS_, ZnO_comm_/PC50, and ZnO_SAS_/PC50 samples under UV irradiation and using ZnO_comm_/PC50 and ZnO_SAS_/PC50 composites under visible irradiation.

Sample	k_UV_ [min^−1^] * UV Light Tests	k_Vis_ [min^−1^] ** Visible-Light Tests
PC50	1.18	-
ZnO_comm_	0.40	-
ZnO_SAS_	0.32	-
ZnO_comm_/PC50	1.43	0.0085
ZnO_SAS_/PC50	2.00	0.0131

* kUV calculated after 10 min of UV LED irradiation time. ** kVis calculated after 180 min of visible LED irradiation time.

## Data Availability

Data will be made available upon request.
